# Urinary proteomic and non-prefractionation quantitative phosphoproteomic analysis during pregnancy and non-pregnancy

**DOI:** 10.1186/1471-2164-14-777

**Published:** 2013-11-11

**Authors:** Jianhua Zheng, Liguo Liu, Jin Wang, Qi Jin

**Affiliations:** 1 MOH Key Laboratory of Systems Biology of Pathogens, Institute of Pathogen Biology, Chinese Academy of Medical Sciences & Peking Union Medical College, No.6, Rongjing East Street, BDA, Beijing 100176, China

**Keywords:** Proteomic profile, Quantitative phosphoproteomic analysis, Human urine, Pregnancy and non-pregnancy, mTRAQ labeling

## Abstract

**Background:**

Progress in the fields of protein separation and identification technologies has accelerated research into biofluids proteomics for protein biomarker discovery. Urine has become an ideal and rich source of biomarkers in clinical proteomics. Here we performed a proteomic analysis of urine samples from pregnant and non-pregnant patients using gel electrophoresis and high-resolution mass spectrometry. Furthermore, we also apply a non-prefractionation quantitative phosphoproteomic approach using mTRAQ labeling to evaluate the expression of specific phosphoproteins during pregnancy comparison with non-pregnancy.

**Results:**

In total, 2579 proteins (10429 unique peptides) were identified, including 1408 from the urine of pregnant volunteers and 1985 from the urine of non-pregnant volunteers. One thousand and twenty-three proteins were not reported in previous studies at the proteome level and were unique to our study. Furthermore, we obtained 237 phosphopeptides, representing 105 phosphoproteins. Among these phosphoproteins, 16 of them were found to be significantly differentially expressed, of which 14 were up-regulated and two were down-regulated in urine samples from women just before vaginal delivery.

**Conclusion:**

Taken together, these results offer a comprehensive urinary proteomic profile of healthy women during before and after vaginal delivery and novel information on the phosphoproteins that are differentially regulated during the maintenance of normal pregnancy. Our results may provide a better understanding of the mechanisms of pregnancy maintenance, potentially leading to the development of biomarker-based sensitive assays for understanding pregnancy.

## Background

Urine is formed via the filtration of plasma by glomeruli in the kidneys, which act as a filter to retain most of the proteins present in the plasma [1]. Because urine collection is non-invasive, low in cost and convenient and because urine is stable compared with other biofluids, urine is advantageous for various clinical applications, such as disease diagnosis, prognosis and guidance of treatment [2]. From a proteomic standpoint, urine can provide proteins for proteomic profile of specific physiological conditions, such as pregnancy, that may be suitable for clinical applications [3]. The urinary proteome has been investigated in several previous studies that have employed different approaches, particularly involving developments in mass spectrometry (MS) with high resolution and accuracy at both the MS and MS/MS levels [4-9].

As it is well known, pregnancy affects protein expression in maternal serum and urine. Furthermore, quantitative differences in protein expression have been detected during pregnancy, which have been useful for the detection of biomarkers for pregnancy-related conditions, such as the identification of fetuses with Down syndrome and preeclampsia, among others [10]. Protein phosphorylation is a dynamic and reversible modification involved in most cell regulatory processes [11]. The determination of the stoichiometry of phosphorylation at certain sites is helpful to understand the mechanism of certain regulatory pathways [12]. Therefore, appreciable efforts have been made toward the analysis of the proteome and phosphoproteome. Analysis of phosphopeptides requires their specific enrichment from unmodified peptides, as well as their fractionation, which reduces the sample complexity. Unmodified peptides tend to elute in the flow-through using methods that selectively enrich the concentration of modified peptides [13]. Recently, a method using a nano-liquid chromatography (LC) configuration featuring low void volume, highly reproducible chromatographic separation and a long, shallow chromatographic gradient was developed to separate highly complex tryptic peptide mixture without pre-fractionation [14]. The resulting peptides were sufficiently resolved by a 5 h gradient using an LTQ Orbitrap analyzer for protein identification and quantification. This method demonstrates that the combination of long-gradient ultra-high pressure liquid chromatography (UHPLC) with high resolution MS at increased sequencing speeds enables extensive proteomic analysis in single runs [15,16].

In the present study, urine samples from pregnant volunteers in their third trimester (the day just prior to vaginal delivery) and one month after vaginal delivery were separated by 1-D SDS-PAGE. Each lane was cut into 22 bands, and the gel slices were digested with trypsin. The resulting peptides were then separated by reversed-phase (RP) LC and analyzed using a LTQ Orbitrap Velos to improve the identification coverage and reliability. In total, 2579 proteins, including 1408 from the urine of pregnant volunteers and 1985 from the urine of non-pregnant volunteers, were identified. Furthermore, using mTRAQ labeling followed by LC-MS/MS for the quantitative phosphoproteomic analysis, 237 phosphopeptides were detected, representing 105 phosphoproteins. Statistical significance was determined using a Student’s t-test and 23 phosphopeptides corresponding to 16 proteins were found to be significantly differentially expressed in the urine of pregnant volunteers. Taken together, the results of this study represent the most comprehensive proteomic characterization of human urine to date. The use of quantitative proteomic approaches to investigate changes in phosphoprotein expression could provide insights into the mechanisms of pregnancy maintenance; potentially contributing to the development of specific and sensitive diagnostic strategies for pregnancy-related conditions.

## Results

### Identification of urinary proteins

The proteome of urinary samples collected just prior to and one month after vaginal delivery from 10 pregnant women was analyzed and compared using a combined SDS-PAGE/LC-MS/MS. Because the normal total protein concentration in urine is very low, various sample preparation procedures, including centrifugation, dialysis, affinity enrichment and precipitation using organic solvents, have previously been used [6]. Here we used a 3 kDa membrane ultrafiltration unit to minimize protein loss and, more importantly, remove low molecular weight polypeptides, which are abundant in human urine samples [17]. The urine samples were concentrated approximately 100-fold using the ultrafiltration unit and then treated with sodium deoxycholate and precipitated with trichloroacetic acid (TCA) to concentrate the urine proteins. As this approach enabled us to treat the urine samples in a large volume and reduce the concentration time, protein degradation was consequently decreased [9]. The concentrated proteins from the pooled samples were then separated by SDS-PAGE, and the gel was cut into 22 bands (lane 1 and lane 2, respectively). Digests from each band were desalted and analyzed by LC-MS/MS.

From the pooled urine samples from pregnant women obtained just before vaginal delivery, we obtained 5355 unique peptides, representing 1408 proteins. For the urine samples obtained one month after vaginal delivery from the same women, we unambiguously identified 8641 unique peptides, representing 1985 proteins. In total, from the urine samples obtained from these women before and after vaginal delivery, we obtained 2579 proteins (10429 unique peptides) after the removal of redundant proteins. On average, more than 4 peptides were used to identify each protein, and the amino acid sequence coverage was approximately 13.7%.

**Figure 1:**
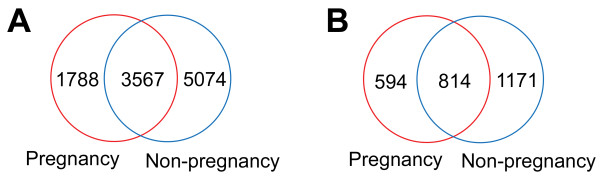
**Venn diagram indicating the overlapping identification of urine samples from pregnant and non-pregnant patients. (A)** The distribution of identified unique peptides. **(B)** The distribution of all identified proteins with at least one unique peptide per protein.

Interestingly, 3567 peptides representing 814 proteins were identified in urine samples of women both before (pregnant) and after (non-pregnant) vaginal delivery (Additional file 1: Table S1b and S1c.). Additionally, 1788 and 5074 peptides were unique to pregnant and non-pregnant women, respectively, while 594 and 1171 proteins were unique to these groups, respectively. Figure 1 indicates the distribution of the peptides and proteins identified from the two sample sets. The complete list of peptides and proteins identified in the present study is provided in Additional file 1: Table S1a.

### Molecular weight and p*I* distributions of the identifications

**Figure 2:**
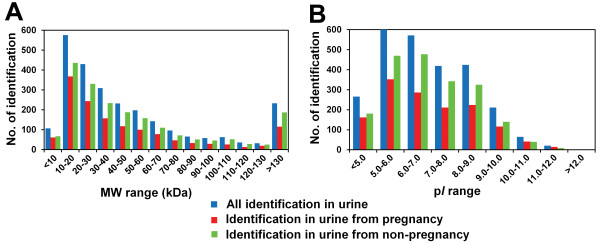
**Numbers of total protein identified in this study, including identification from pregnant and non-pregnant patients.** The distributions are in different **(A)** molecular weight (MW) range and **(B)** p*I* range. All identified proteins are illustrated in the blue histogram, while the identifications from pregnant and non-pregnant patients are in red and green, respectively.

The theoretical M*r* distribution for the identified proteins ranged from 1.5 kDa to 2991.1 kDa (TTN) and is depicted in Figure 2A. The majority of the proteins were in the range between 10 and 70 kDa, representing approximately 73.2% (1888 of 2579) of all the identified proteins. The molecular weight distribution of the urinary proteins during pregnancy was similar to that during non-pregnancy. Interestingly, 233 proteins with a relative high molecular weight (more than 130 kDa) were detected in this study, indicating that the identification of high molecular weight proteins may benefit from a thorough gel separation. In contrast, only 4.1% (107 of 2579) of the low molecular weight proteins (less than 10 kDa) were identified. Because proteins with higher molecular weight may generate more peptides than lower weight proteins after digestion with trypsin, it is presumed that the latter might be masked by the former [18].

The p*I* scores for the identified proteins ranged from 4.1 (DPH3) to 12.5 (C10orf140), and a detailed p*I* distribution is illustrated in Figure 2B. Out of all the identifications, it was clear that the majority of the proteins clustered around a p*I* of 5–9, which is similar to the total proteome. There were only two proteins with p*I* scores over 12.

### Functional classification of the identifications

**Figure 3:**
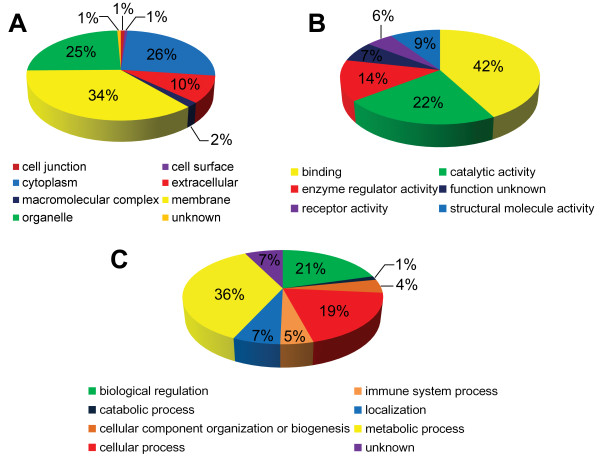
**Distribution of the proteins based on Gene Ontology analysis, including (A) cellular component, (B) molecular function and (C) biological process using the Gene Ontology (**http://www.geneontology.org**).** The compositions of the protein categories are presented as percentages of all identified proteins.

The proteins identified were classified according to Gene Ontology. The classification based on cellular component (Figure 3A) revealed that the majority of the proteins are known to be either plasma membrane (34%) or present on the cytoplasm (26%). We also identified a number of organelle proteins (25%) that are known to be on nucleus (3%), lysosomal lumen (2%), mitochondrion (2%), Golgi apparatus (2%) and vesicular exosome (2%) et al. In terms of molecular function (Figure 3B), the majority of the proteins are categorized into groups involved in binding (42%), catalytic activity (22%), enzyme regulator activity (14%), structural molecule activity (9%) and receptor activity (6%). In binding group, the proteins were involved in ion binding (16%), protein binding (9%), ATP and GTP binding (4%), carbohydrate binding (2%) and DNA binding (1%) et al. In catalytic activity group, the proteins were involved in hydrolase activity (9%) and oxidoreductase activity (5%) and serine-type endopeptidase activity (5%) et al. The proteins were also classified based on biological process into those involved in metabolic process (36%), biological regulation (21%), cellular process (19%), localization (7%), cellular component organization or biogenesis (5%) and immune system process (4%). Additionally, 7% of the proteins were unknown proteins (Figure 3C).

### Comparison with other studies on urinary proteomes

A numbers of studies have been performed to characterize the urinary proteome in healthy individuals [2-9,19-26]. Table 1 summarizes the major studies carried out to date. By comparing our results with previous urinary proteomic studies, we found that 1556 of the proteins identified here overlapped with those in previous studies. It should be noted that inter-individual differences likely account for some of the observed variability in the proteomes, particularly for urine samples during pregnancy and non-pregnancy, and thus the overlap is relatively low. Therefore, there are 1023 proteins not reported by the others studies at the proteome level that are unique to our study. These unique proteins were indicated in blue font in Additional file 1: Table S1d.

**Table 1:** **Major proteomic studies performed on human urine**^**a**^

**Year**	**Title**	**Sample**	**No. of identifications**	**Analysis method and instrument used**	**Reference**
2004	Identification and proteomic profiling of exosomes in human urine	exosome	295	1-DE and LC–MS/MS	[25]
2004	Establishment of a near-standard two-dimensional human urine proteomic map	urine	113	2-DE and MALDI TOF	[22]
2005	Exploring the hidden human urinary proteome via ligand library beads	urine	383	2-DE and SELDI-TOF; LC FT-ICR	[8]
2005	Human urine proteome analysis by three separation approaches	urine	226	1-DE and 2D LC LCQ-DECA^Xp^plus	[24]
2005	Development of a high-throughput method for preparing human urine for two-dimensional electrophoresis	urine	50	2-DE and 4700 TOF/TOF MS	[21]
2006	The human urinary proteome contains more than 1500 proteins, including a large proportion of membrane proteins	urine	1543	1-DE and LC LTQ-Orbitrap and LC-FT	[6]
2006	Characterization of the human urine proteome by preparative electrophoresis in combination with 2-DE	urine	141	2-DE and MALDI TOF	[20]
2006	Simple urinary sample preparation for proteomic analysis	urine	339	2-DE and MALDI TOF	[9]
2008	Optimizing sample handling for urinary proteomics	urine	735	1-DE and LC LTQ	[4]
2009	Urine proteomics for profiling of human disease using high accuracy mass spectrometry	urine	2362	1-DE and LC LTQ-Orbitrap	[5]
2009	Large-scale proteomics and phosphoproteomics of urinary exosomes	exosome	1132	1-DE and LC LTQ	[26]
2009	High speed two-dimensional protein separation without gel by isoelectric focusing-asymmetrical flow field flow fractionation: application to urinary proteome	urine	245	2D-IEF and LC LCQ-Deca^XP^	[23]
2010	A comprehensive and non-prefractionation on the protein level approach for the human urinary proteome: touching phosphorylation in urine	urine	1310	2D LC LTQ-Orbitrap	[2]
2011	A comprehensive map of the human urinary proteome	urine	1823	1-DE and LC LTQ-Orbitrap Velos	[3]
2012	Analysis of the urine proteome via a combination of multi-dimensional approaches	urine	558	1-DE and quadrupole ITMS	[19]
2012	Urine proteome of autosomal dominant polycystic kidney disease patients	urine	1700	IEF and LC LTQ-Orbitrap Velos	[7]
2013	Urinary proteomic and non-prefractionation quantitative phosphoproteomic analysis during pregnancy and non-pregnancy	urine	2579	1-DE and ESI-MS/MS (LTQ-Orbitrap Velos)	this study

^a^Summary of the major mass-spectrometry-based studies performed on the urinary proteome is listed. Table includes details about the numbers of total proteins, sample categories and the analysis method and the type of mass spectrometer used in the listed studies.

### Phosphoproteomic analysis of urine samples

Recently, technical approaches that allow for phosphoproteomic profiling with long gradient LC-MS have been introduced [14,15]. We used this strategy to identify phosphoproteins and phosphorylation sites present in urine samples. In total, 237 phosphopeptides (130 unique phosphopeptides) with 222 phosphorylation sites were confidently identified, representing 105 phosphoproteins (see Additional file 2: Table S2). Of the unique phosphopeptides, 62% were mono-phosphorylated peptides, 23% were di-phosphorylated peptides and 15% were multi-phosphorylated (more than two) peptides. Interestingly, five phosphopeptides had six phosphorylation sites. Additionally, of the 222 phosphorylation sites, 70% (156 of 222) were phosphorylated at serine, 22% (49 sites) at threonine and 8% (17 sites) at tyrosine residue. The distribution of phospho-amino acids in the present study is consistent with other urinary proteomic studies [2].

For the mTRAQ-based quantitative phosphoproteomic analysis, the phosphopeptides that passed t-test with p-value < 0.05 and log2 fold change values ≥0.6 or ≤ −0.6 (increasing or decreasing 1.5 fold in phosphopeptide) were considered to be significantly regulated. Therefore, average normalized ratios were used for the proteins ratios, and a cutoff value of 1.5-fold was chosen as the threshold for screening significantly changed proteins. In total, 23 unique phosphopeptides corresponding to 16 proteins were found to be differentially expressed, of which 14 were up-regulated and two were down-regulated in urinary samples from women just before vaginal delivery. Additional file 3: Table S3 shows the phosphoproteins corresponding to peptides that were significantly altered.

## Discussion

Recent advances in proteomics, particularly developments in MS with high resolution and accuracy levels, have allowed for unprecedented discovery of the composition of human urine and the application of this knowledge to the study of human physiology and disease biomarker. In the current study, we used urine samples to understand protein expression patterns and the maintenance of these expression patterns during pregnancy compared with the non-pregnant state. In contrast to conventional biomedical approaches, which can only monitor one or a small number of specific proteins at a time, we combined extensive gel fractionation with high resolution and accuracy LTQ Orbitrap-MS/MS, which can measure protein expression levels directly and provide insight into the activity state of all of relevant proteins under different physiological conditions.

Initially, we constructed a protein profile map of urine obtained from ten healthy pregnant women and the same non-pregnant women following delivery. Protein identification data reported here is the largest catalog of proteins from the urinary proteome identified in a single study to date. We believe that this result is likely the result of employing multiple gel pre-fractionations (twenty-two strips each lane), which decreased the complexity of the sample [27]. Furthermore, we used a LTQ Orbitrap Velos with high resolution and accuracy settings at both the MS and MS/MS levels, while many of the earlier studies were performed on other instruments with lower resolutions and sensitivities. Additionally, we integrated CID and HCD fragmentation mode MS analysis to give the highest number of peptide identifications [28].

Different proteins observed in urine samples are likely due to differences physiologies state of pregnancy and non-pregnancy. In agreement with previous studies, we observed a relative enrichment of the urinary proteome with respect to different physiologies in pregnancy versus non-pregnancy. A total of 594 proteins were found to be unique to pregnant women. These unique proteins may provide insight into the biomarkers present under pregnant conditions. For example, annexins are known to be abundant in placental membranes; some of these proteins were detectable during pregnancy with a 4-fold peak change in expression. The high expression of annexin 2 was maintained during pregnancy [29]. Therefore, usage of advanced MS methods for the study of human urine promises to offer significant insights into human physiology and its application to the diagnosis of pregnancy.

To date, little information on the phosphoproteomic changes that occur during pregnancy has been obtained. Furthermore, most previous studies did not follow the changes over time from pre- to post-vaginal delivery as we have performed here. The aim of this study was to investigate the total protein expression patterns and phosphoprotein expression changes just before and after vaginal delivery and to identify the proteins that are important for maintaining pregnancy. For each mTRAQ experiment, we also combined CID and HCD data to derive a set of unique phosphopeptides. In addition to providing quantitative information (via mTRAQ reporter ions), HCD fragmentation also gave a significant number of unique phosphopeptides [30]. As a result, however, the amount of material available for each condition (pregnancy and non-pregnancy) was limited to approximately 100 μg according to the manufacturer’s protocol (see Materials and methods for details), resulting in a total of approximately 400 μg of labeled peptides after pooling the two samples into a single mTRAQ set. This is a significant departure from conventional phosphoproteomic studies, which typically involve large amounts of starting material (up to 10 mg), thus allowing for extensive peptide fractionation in MS analysis. Hence, our experimental approach was constrained to phosphopeptide enrichment followed by a mTRAQ labeling strategy due to the limited samples availability. Although some single phosphoproteomic analyses using urinary samples have been published [2,26,31], no phosphoproteomic differential expression analyses employing highly specialized states (e.g. just before and after vaginal delivery) have been conducted to our knowledge. Thus, our data represent one of the first large-scale phosphoproteomic studies using urine samples from healthy women before and after vaginal delivery.

The number of unique phosphopeptides, phosphoproteins and phosphorylation sites identified in this study was particularly impressive considering the limited amount of starting material. Some of these phosphoproteins are pivotal in the maintenance of pregnancy and may play diverse functional roles. For instance, Alpha-2-HS-glycoprotein (AHSG) is a 49 kDa protein that plays a role in host defense and bone metabolism [32]. This protein is one of the major components of the non-collagenous bone matrix, particularly in the fetuses. AHSG must be phosphorylated to be physiologically active in mammals, and it has been shown that the non-phosphorylated version exerts minimal or no effects [33]. Three distinct phosphorylated peptides and two phosphorylation sites, Ser204 and Ser394, were found in the present study (see Additional file 2: Table S2). Compared with samples from non-pregnant women (after vaginal delivery), samples from women in their third trimester of pregnancy had AHSG concentrations that were significantly increased (over 26-bold higher). Additionally, it was reported that the concentration of this protein was also significantly higher in the third trimester compared with the first and second and with the non-pregnant controls [34]. It is tempting to speculate on the biological significance of this protein in pregnancy, during which its concentration increases. Another key factor, secreted protein acidic and rich in cysteine-like protein 1 (SPARCL1), an extracelluar matrix glycoprotein, is implicated in many physiological functions [35]. SPARCL1 is overexpressed in many tumors of the digestive tract [36], and Turtoi et al. reported that SPARCL1 is a new marker of human glioma progression [37]. Although SPARCL1 levels are low in the placenta [36], a significant increase (7-bold higher) of this protein in urine was observed during the third trimester of pregnancy compared to non-pregnancy. Here three unique phosphorylated peptides and three phosphorylation sites were found to map to the protein (see Additional file 2: Table S2). Interestingly, all of the phosphorylation sites were on serine residues (Ser92, Ser198 and Ser295). Additionally, secreted phosphoprotein one (SPP1), also known as osteopontin (OPN), is an acidic single chain phosphorylated glycoprotein component of the extracellular matrix [38]. Human urine contains different forms of OPN, which is subjected to significant posttranslational modifications, such as phosphorylation, sulfation and glycosylation [39]. The intact protein in urine contains approximately eight phosphate groups distributed over 30 phosphorylation sites. Here, among twenty distinct phosphorylation sites detected in the present study, Ser254 and Ser275 were consistent with the above report [39], whereas the additional Ser62 was previously described in human milk [40]. Thirteen phosphosites (Ser62, Ser63, Ser224, Thr227, Ser234, Ser254, Ser258, Ser263, Ser270, Ser275, Ser303, Ser308 and Ser310) were also reported elsewhere in urine [2]. Finally, seven novel phosphosites (Ser24, Ser26, Ser27, Ser191, Ser195, Tyr202 and Ser280) were detected for the first time in this study.

Other phosphoproteins, such as insulin-like growth factor-binding protein 1 (24-bold higher), liver-expressed antimicrobial peptide 2 (12-bold higher), three prime repair exonuclease 1 (10-bold higher) and high affinity copper uptake protein 1 (5-bold higher), were also found to be significantly altered in this study. These differentially expressed phosphoproteins might participate in maintaining a successful normal pregnancy.

## Conclusion

Our results represented a comprehensive proteomic profile and the phosphoprotein expression pattern of urine samples during before and after vaginal delivery. A subset of the identified proteins may have important roles in maintaining a normal, successful pregnancy and may be useful for preventing pregnancy failure by comparing a patient’s expression profile with those under pathological conditions known to cause pregnancy failure. Although we have only identified a limited number of phosphoproteins (105 total phosphoproteins and 16 differentially expressed phosphoproteins), these preliminary data present the possibility for a phosphoproteomic study and an evaluation of the significance of the protein changes observed during pregnancy. Further study will be needed to elucidate the specific involvement of differentially expressed proteins during pregnancy, and certainly, other supplementary methods will be needed to confirm these findings.

## Methods

### Sample collection and preparation

Ten healthy women (aged 25 to 30) were recruited for the study. Informed consents were obtained from all subjects and the study was reviewed and approved by the Ethics Committee of the Institute of Pathogen Biology, Chinese Academy of Medical Sciences & Peking Union Medical College. Urine samples from pregnant and nonpregnant women were collected by Beijing Tongren Hospital, China. Fifty milliliters of morning midstream urine were collected from each individual one day prior to and 30 days after vaginal delivery (In Chinese, this period is literally referred to as “sitting the month”), respectively. Equal volumes of urine samples from the 10 individuals were pooled, and protease and phosphatase inhibitors (Roche, Germany) were added to prevent protein enzymatic breakdown or modification prior to processing. The two urine sample sets (pregnancy and non-pregnancy) were prepared as previously described with modifications [3]. Briefly, after centrifugation and filtration, the filtered urine from each set was concentrated using 3 kDa cutoff filters (Millipore, Billerica, MA). The resulting concentrate was treated with 0.015% (w/v) sodium deoxycholate under shaking for 10 min at room temperature (RT), and subsequently subjected to TCA (10%, v/v) precipitation procedure. The resulting solution was incubated 2 hours at −20°C and then centrifuged at 4000 *g* for 15 min to collect the precipitates. After being washed thrice with ice-cold acetone and allowed to air dry, the protein content of the precipitates was quantitated using a bicinchoninic acid protein assay.

### In-gel digestion for LC-MS/MS

Proteins from each sample set were divided into two aliquots. One aliquot was for quantitative phosphoproteome analysis (described below) and the other (10 μg) was suspended in loading buffer and subjected to 12% SDS-PAGE (1.0 mm thick with a width/length of 8.6/6.8 cm). Each lane was cut into 22 bands and subjected to an in-gel tryptic digestion protocol as previously described [41]. All of the tryptic peptides were desalted using ZipTipC18 (Millipore, Billerica, MA), dried under vacuum and solubilized in 0.1% formic acid for subsequent LC-MS/MS analysis. In total, we performed 132 RP LC-MS/MS runs (22 bands each lane, with two lanes for urine samples from pregnant and non-pregnant women performed in triplicate).

### In-solution digestion, mTRAQ labeling and phosphopeptide enrichment

One aliquot containing 200 μg of protein from the above sample was reduced, cysteine blocked and in-solution digested according to the manufacturer’s protocol (mTRAQ® Reagents Kit, AB Sciex, Foster City, CA). For each sample set digest (pregnancy and non-pregnancy), on half (containing 100 μg of protein) was transfer to a fresh tube labeled with mTRAQ Reagent Δ8 for the global internal standard. The other half (also containing 100 μg of protein) was labeled with mTRAQ Reagent Δ0 (urine sample from pregnancy) or Δ4 (urine sample from non-pregnancy) for the analytical mixture. The four labeling reactions were then combined, desalted using a OASIS HLB Cartridge column (HLB-3 cc, Waters, Milford, MA). All of the eluted peptide fractions were concentrated with a vacuum centrifuge and solubilized in 200 μl TiO_2_ phosphobind buffer containing 50 g/L 2,5-dihydroxybenzoic acid for subsequent phosphopeptide enrichment using a Phosphopeptide Enrichment TiO_2_ kit (Calbiochem, San Diego, CA) according to the manufacturer’s instruction with slight modifications. Briefly, the tryptic digest was mixed with 50 μl TiO_2_ phosphobind resin and incubated for 1 h. After three washes, the phosphopeptides were eluted twice with 0.5% ammonium solution (pH10.5) in 50% acetonitrile (ACN). The elutions were combined and dried using a vacuum centrifuge and reconstituted in 20 μl of 0.1% formic acid (FA) for LC − MS/MS analysis.

### LC-MS/MS analysis

In-gel digested peptide mixtures were analyzed using a nanoAcquity ultra-performance liquid chromatography (UPLC) system (Waters, Milford, MA) coupled to a high-resolution LTQ Orbitrap Velos mass spectrometer (Thermo Fisher Scientific, Germany), as previously described with slight modifications [41]. A normalized collision energy of 35% and 40% was used for CID and HCD fragmentation, respectively. Up to 20 and 10 most intense precursor ions from the full scan were selected for fragmentation by CID and HCD, respectively. Lock mass calibration using a background ion from the air (*m/z* 445.12003) was applied. In total, we performed 88 RP LC-CID MS/MS runs (two runs using CID fragmentation per fraction, 22 fractions each sample set (pregnancy and non-pregnancy)) and 44 LC-HCD MS/MS runs (one run using HCD fragmentation per fraction).

For the labeled phosphopeptides, a long chromatographic gradient was developed to separate the complex peptide mixture [16]. Peptides were eluted using a 600-min gradient with aqueous solvents as described above. During the elution step, the percentage of solvent B increased in a linear fashion from 5% to 25% at 10–450 min, followed by an increase to 35% at 450–570 min and a column wash at 90% at 571–585 min and re-equilibration at 1% B at 586–600 min. We performed three LC-CID and LC-HCD runs for the phosphopeptide identification.

### MS data processing and analysis

The raw data from in-gel digestions were processed using the Proteome Discovery software (version 1.3.0.339; Thermo Fisher Scientific, Germany) with the search algorithm SEQUEST (Thermo Fisher Scientific, Germany) against the Human IPI database (version 3.87, http://www.ebi.ac.uk/IPI/). Enzyme specificity was set to trypsin/P, and a maximum of two missed cleavages were allowed. Cysteine carbamidomethylation was used as a fixed modification; methionine oxidation and N-terminal acetylation were used as variable modification. The initial maximal allowed mass tolerance was set to 5 ppm for precursor masses and then was set to 0.8 Da for fragment ion masses.

The raw data from the labeled phosphopeptides were processed using MaxQuant software [42] (version 1.2.2.5) with the search engine Andromeda against the Human IPI database. The initial precursor mass tolerance was set to 6 ppm, and fragment ion mass tolerance was set to 0.5 Da for CID MS/MS spectra and 20 ppm for HCD MS/MS spectra. Analysis was limited to peptides of six or more amino acids and maximum two missed cleavages. Carbamidomethyl cysteine was set as a fixed modification and oxidized methionine, N-terminal acetylation and phosphorylated serine, threonine and tyrosine were set as variable modification. The mTRAQ reporter ion intensities were normalized and reporter ion intensities for all spectra identifying the same protein were summed in a given replicate. In the case that identified peptides were shared by two or more proteins (homologs or isoforms), they were reported by MaxQuant as one protein group. The reverse database search option was enabled in above raw data processing, and a maximum target-decoy-based false discovery rate (FDR) of 1.0% for peptide and protein identification was allowed. Furthermore, if standard deviation (SD) of phosphorylation ratios for normalized peptides were greater than 1, quantitation event would also be excluded from further analysis. Moreover, a Student’s t-test was performed using the standard deviation of the pooled sample across the biological replicates and the difference between the control (non-pregnancy) and variable sample (pregnancy) to account for the global sample variability. Given the distributions of measured intensities and ratios of all quantified peptides we calculated a p-value for each medium to light ratio indicating its significance and derived p-values were further corrected for multiple hypothesis testing using Student’s t-test. The phosphopeptides that passed t-test with p-value < 0.05 were considered to be significantly regulated. To increase the confidence of the quantitative data, we also included the cutoff for the log2 fold change values, in which the phosphorylation changes were considered highly significant if the log2 value ≥0.6 or ≤ −0.6 (increasing or decreasing 1.5 fold in phosphopeptide). Additionally, for three repeated protein quantification, average normalized ratios were used for the proteins ratios, and a cutoff value of 1.5-fold was chosen as the threshold for screening significantly changed proteins. The theoretical molecular mass and p*I* value of all the proteins were predicted using ProtParam (http://us.expasy.org/tools/protparam.html). GO analysis was conducted for all the proteins identified in the context of their biological process, molecular function and cellular compartment (http://www.geneontology.org).

### Availability of supporting data

All the raw mass spectra files in LC-MS/MS have been deposited into the publicly accessible database PeptideAtlas and now are available with dataset Identifier PASS00247 (http://www.peptideatlas.org/PASS/PASS00247).

## Competing interests

The authors declare that they have no competing interests.

## Authors’ contributions

JZ and QJ conceived and designed the experiments and carried out the proteomic experiments. LL prepared protein samples and performed quantitative phosphoproteomic experiments. JW participated in the bioinformatic data analysis. JZ prepared and wrote the manuscript. All authors read and approved the final manuscript.

## Supplementary Material

Additional file 1: Table S1 List of 2579 proteins corresponding to peptides identified by LC-MS/MS analysis of the urine samples from pregnant and non-pregnant patients. Identifications in urine samples from both pregnant and non-pregnant patients are illustrated in red font. The 1023 proteins not reported by the others studies at the proteome level were unique to our study. These unique proteins are indicated in blue font. Click here for file

Additional file 2: Table S2 List of 105 phosphoproteins corresponding to phosphopeptides and phosphosites identified by quantitative phosphoproteomic analysis of the urine samples from pregnant and non-pregnant patients. Click here for file

Additional file 3: Table S3 List of the 16 phosphoproteins differentially expressed in the urine samples from pregnant and non-pregnant patients. For three repeated protein quantification, average normalized ratios were used for the proteins ratios, and a cutoff value of 1.5-fold was chosen as the threshold for screening significantly changed proteins. Click here for file
